# A comprehensive analysis of NPHS1 gene mutations in patients with sporadic focal segmental glomerulosclerosis

**DOI:** 10.1186/s12881-019-0845-4

**Published:** 2019-06-19

**Authors:** Ling Zhuo, Lulin Huang, Zhenglin Yang, Guisen Li, Li Wang

**Affiliations:** 1 Renal Department and Institute of Nephrology, Sichuan Provincial People’s Hospital, School of Medicine, University of Electronic Science and Technology of China, No. 32, West 2nd Duan, 1st Circle Road, Qingyang District, Chengdu, Sichuan 610072 People’s Republic of China; 2 Key Laboratory for Human Disease Gene Study, Sichuan Provincial People’s Hospital, School of Medicine, University of Electronic Science and Technology of China, Chengdu, 610072 China

**Keywords:** Focal segmental glomerulosclerosis, NPHS1, Mutation, Second-generation sequencing

## Abstract

**Background:**

Focal segmental glomerulosclerosis (FSGS) is still one of the common causes of refractory nephrotic syndrome. Nephrin, encoded by podocyte-specific NPHS1 gene, participated in the pathogenesis of FSGS. The sites of NPHS1 mutations in FSGS is not clarified very well. In this study, we investigated the specific mutations of NPHS1 gene in Chinese patients with sporadic FSGS.

**Methods:**

A total of 309 patients with sporadic FSGS were collected and screened for NPHS1 mutations by second-generation sequencing. The variants were compared with those extracted from 2504 healthy controls in the 1000 Genomes Project. The possible pathogenic roles of missense variants were predicted by three different software. We also compared these candidate causal mutations with those summarized from the previous studies.

**Results:**

Thirty-two genetic mutations of NPHS1 gene were identified in FSGS patients, including 12 synonymous mutations, 17 missense mutations, 1 splicing mutation, and 2 intron mutations, of which c.G3315A (p.S1105S) was the most common variant (261/309). A novel missense mutation c.G2638 T (p.V880F) and a novel splicing mutation 35830957 C > T were identified in FSGS patients. The frequencies of the four synonymous mutations (c.C294T [p.I98I], c.C2223T [p.T741 T], c.C2289T [p.V763 V], c.G3315A [p.S1105S]) were much higher in FSGS patients than in controls. The frequencies of the four missense mutations (c.G349A [p.E117K], c.G1339A [p.E447K], c.G1802C [p.G601A], c.C2398T [p.R800C]) were much higher and one (c.A3230G [p.N1077S]) was lower in FSGS patients than in controls. Five missense mutations, c.C616A (p.P206T), c.G1802C (p.G601A), c.C2309T (p.P770L), c.G2869C (p.V957 L), and c.C3274T (p.R1092C), were predicted to be pathogenic mutations by software analysis.

**Conclusions:**

NPHS1 gene mutations were quite common in sporadic FSGS patients. We strongly recommend mutation analysis of the NPHS1 gene in the clinical management of FSGS patients.

## Background

Focal segmental glomerulosclerosis (FSGS) is a syndrome with unique clinical and pathological manifestations. Although new medications continue to emerge, FSGS is still one of the most common causes which contributes to refractory nephrotic syndrome [1–3]. The previous studies suggested that FSGS could be idiopathic or secondary to a process originating outside the kidneys as well as to a specific genetic mutation [1, 3, 4], including INF2, ACTN4, TRPC6, WT1, NPHS1, NPHS2, etc. [5, 6]. The efficacy of cyclosporin A (CsA) is much better in nonhereditary steroid-resistant nephrotic syndrome (SRNS) than in genetic SRNS [7]. It suggests that genetic mutations not only contribute to the pathogenesis of FSGS but also influence the outcome of clinical treatment. Therefore, it is of great clinical significance to elucidate the characteristics of genetic variation for FSGS patients.

About 8–14% of patients with FSGS could be explained by podocyte-related gene mutations. NPHS1, that relates to congenital nephrotic syndrome (CNS) and SRNS, is one of the most frequently reported genes. Initially, Kestilä described NPHS1 as the pathogenic gene of the congenital nephrotic syndrome of the Finnish type (CNF) [8]. The NPHS1 gene (OMIM *602716) locates in the chromosome 19q13.1 and consists of 29 exons that span a 150 kb region. Nephrin, a transmembrane protein encoded by NPHS1 gene, is one of the important components of slit diaphragm (SD) [9]. As a signaling scaffold via interactions at its short intracellular region, nephrin also serves as the core component of the glomerular filtration barrier [10]. The mutations of NPHS1 gene could lead to the occurrence of different degrees of kidney diseases, however, studies which investigated the relationship between NPHS1 gene mutations and FSGS were limited, especially in China [8, 11–13].

Santin et al selected SRNS patients to conduct a series of research of podocyte-associated genes [6]. The patients showed familiar heredity and 57% of them developed FSGS at extremely early ages [6]. NPHS1 gene was the most common mutant gene in these subjects, and patients with NPHS1 mutations were more likely to progress to ESRD than those with other podocyte genes [6]. NPHS1 mutations have exhibited increasing prevalence across the world over recent decades, while most of the reports have only focused on one or several mutations of NPHS1 in relatively small FSGS samples. In this study, we performed an analysis of NPHS1 gene mutations in Chinese patients with sporadic FSGS by direct sequencing of all exons. While comparing these variants with the data from 1000 genome project as well as the mutations reported previously, we hoped to further elucidate the characteristics of NPHS1 gene mutations in sporadic FSGS patients and to provide a profile for future precision medicine and pathogenetic studies of FSGS.

## Subjects and methods

### Patient and data recruitment

A total of 309 biopsy-proved FSGS patients were enrolled in this study. All patients with secondary FSGS and familial FSGS were excluded. The study was approved by the South West research ethics committee and the institutional review board at each recruiting center. The average age of the patients was 32.0 ± 14.2 years old (from 10 to 71 years old). The percentage of male patients was 62.0% (195 cases). The NPHS1 mutations information of 2504 healthy controls was extracted from the 1000 Genome Project (International Genome Sample Resource, IGSR, www.internationalgenome.org).

We summarized the data of NPHS1 gene mutations from previous publications. Detailed mutational information in previous references for retrospective analysis was collected from PUBMED database (https://www.ncbi.nlm.nih.gov/pubmed). All the locations of the mutation sites were determined according to the reference sequence of NPHS1 derived from current assembly GRCh38.p11.

### Exome sequencing and variant detection

Genomic DNA was extracted from peripheral blood using standard methods. An exome sequencing for the first 41 FSGS samples was provided by Axeq Technology Inc., Seoul, Republic of Korea. The sequenced sample was prepared according to the Illumina protocols of Sure Select Target Enrichment System Capture Process. Exome sequencing analysis was performed as described previously [14]. The peripheral blood DNA samples of 268 patients with sporadic FSGS were sequenced by next-generation sequencing. Exon regions of NPHS1 gene were selected and the biotinylated 60mer probes were designed to tile along with the exons of the genes. Samples were prepared as an Illumina sequencing library, and in the second step, the sequencing libraries were enriched for the target region related genes using the MyGenostics Target Region Enrichment protocol. The captured libraries were sequenced using Illumina HiSeq 2000 Sequencer.

Then these variants were filtered through a stringent strategy. Firstly, the detected variants were annotated and filtered based on public and in-house databases: (i) variants in dbSNP144 (http://www.ncbi.nlm.nih.gov/projects/SNP/); (ii) 1000 Genomes Project (ftp://ftp.1000genomes.ebi.ac.uk/vol1/ftp); and (iii) ESP6500 (http://evs.gs.washington.edu/EVS). Secondly, possible damaging effect of each variant on protein structure/function was predicted by SIFT [15], PolyPhen2 [16] and MutationTaster [17]. For a nonsynonymous single nucleotide variant (SNV), the predicted results of “Probably damaging”, “Possible damaging”, “Damaging”, as well as “Disease-causing”, were recorded.

### Mutation analysis

All variants located in the total 29 exons and exon-intron boundaries of NPHS1 were analyzed. All statistical tests were performed on SPSS software Version 17.0 (IBM Corp., USA). Chi-square tests in 2 × 2 tables were conducted to compare the mutation distribution between the FSGS patients and healthy controls. Bonferroni-corrected *P* values < 0.05 were considered statistically significant.

### Ethics and consent to participate

This study was approved by Institutional Review Boards of the Sichuan Academy of Medical Sciences and Sichuan Provincial People’s Hospital. All patients provided informed written consent for the collection of data and genetic analysis obtained. Written informed consent from legal guardians of those under the age of 18 was collected.

## Results

### The variant distributions of NPHS1 in FSGS patients

**Fig. 1:**
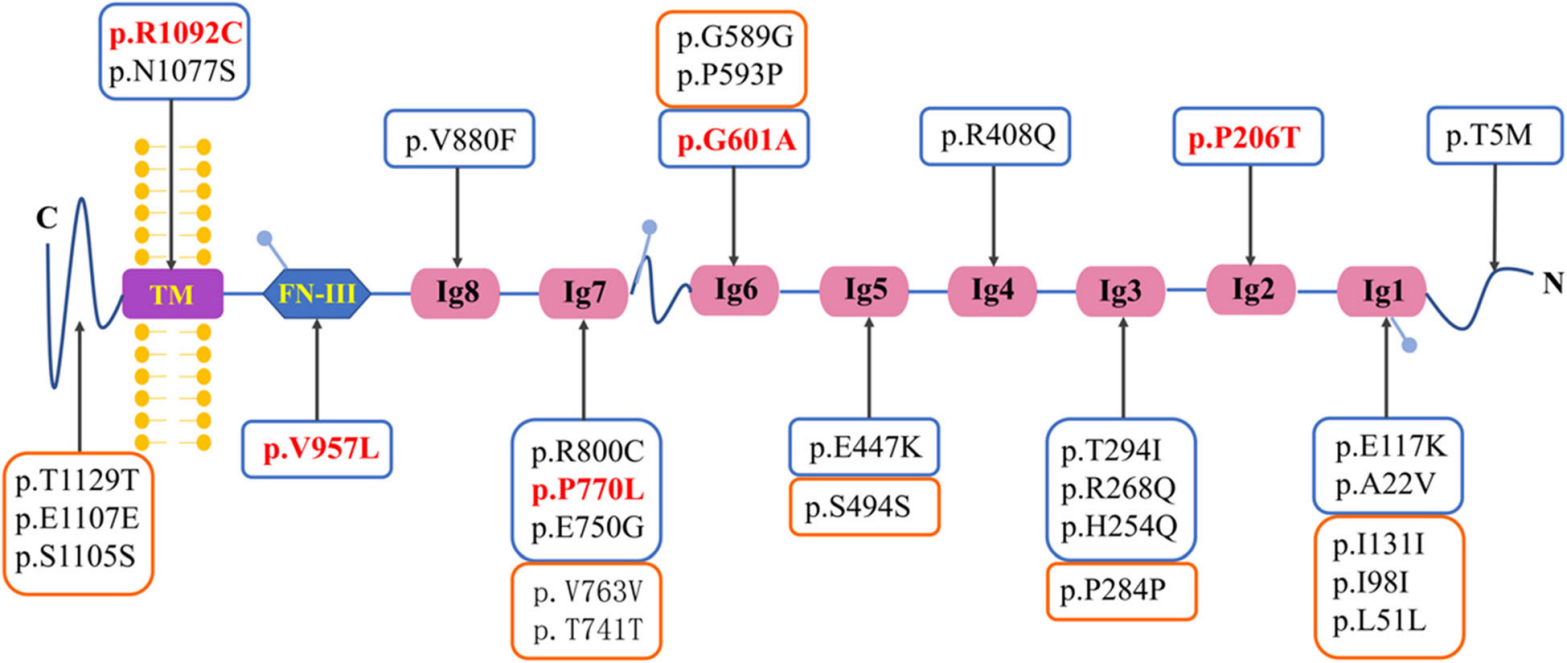
The variant distributions of NPHS1 in the domains of nephrin protein. Nephrin protein consists of a C-terminal cytoplasmic domain (blue curled line-C), a transmembrane domain (purple rectangle, TM), a fibronectin type III-like module (blue rhombus, FNIII), eight extracellular Ig-like domains (pink oval, Ig 1–8) and a signal peptide domain (blue curled line-N). All amino acid changes found in this study were listed. The orange boxes indicate the synonymous mutations for the corresponding protein domains. The blue boxes indicate the missense mutations for the corresponding protein domains. Red bold font indicates the strong susceptibility to mutations

Twenty-two variants of NPHS1 gene were identified in 309 FSGS patients, including 12 synonymous mutations, and 17 missense mutations. Moreover, two mutations were found in introns and one novel splicing mutation 35830957 C > T was found in exon 28. One more novel missense mutation c.G2638 T (p.V880F) was also identified in FSGS patients. All these mutations were annotated in the domains of nephrin protein (Fig. 1). Mutations mostly affected Ig-like domains of the nephrin protein, the extracellular domain, as well as the intracellular domain.

### Synonymous mutations and intron mutations

Twelve synonymous mutations were found in FSGS patients. Among them, only three mutations had been investigated in previous reports (Table 1).

**Table 1:** The synonymous mutations detected in FSGS patients and compared with controls from 1000 Genomes Project

Exon	dbSNP144	Position	Transcript consequence	Protein consequence	Patients(*n* = 309)	Controls(*n* = 2504)	*P* value	Bonferroni-corrected *P* value	Nephrin domain	Initial phenotype
2	rs114385015	35851580	c.C151T	p.L51 L	2((0.65%)	9 (0.36%)	0.35	> 0.05	Ig 1	Unknown
3	rs2285450	35851365	c.C294T	p.I98I	93 (30.10%)	157 (6.27%)	2.35 × 10^−31^	7.51 × 10^− 30^	Ig 1	Unknown
3	rs181246281	35851266	c.C393T	p.I131I	1 (0.32%)	1 (0.04%)	0.21	> 0.05	Ig 1	Unknown
8	rs763233132	35849136	c.G852A	p.P284P	1 (0.32%)	0	0.11	> 0.05	Ig 3	Unknown
12	rs549535993	35846153	c.G1482A	p.S494S	1 (0.32%)	0	0.11	> 0.05	Ig 5	Unknown
14	rs770065180	35845519	c.A1779C	p.P593P	1 (0.32%)	0	0.11	> 0.05	Ig 6	Unknown
14	rs768531638	35845531	c.C1767G	p.G589G	1 (0.32%)	0	0.11	> 0.05	Ig 6	Unknown
17	rs2073901	35843583	c.C2223T	p.T741 T	47 (15.21%)	31 (1.24%)	7.81 × 10^−27^	2.50 × 10^−25^	Ig 7	SRNS [40]
17	rs437168	35843517	c.C2289T	p.V763 V	99 (32.04%)	304 (12.14%)	5.02 × 10^−15^	1.61 × 10^−13^	Ig 7	SRNS [40], MCNS [41]
26	rs115670171	35831362	c.G3321A	p.E1107E	3 (0.97%)	1 (0.04%)	4.82 × 10^−3^	0.16	Cy	Unknown
26	rs780661566	35831296	c.G3387A	p.T1129 T	1 (0.32%)	0	0.11	> 0.05	Cy	Unknown
26	rs2071327	35831368	c.G3315A	p.S1105S	261 (84.47%)	426 (17.01%)	2.20 × 10^−14^	7.02 × 10^−13^	Cy	SRNS [40], MCNS [41]
Intron	rs466452	35831607	c.C3286 + 36 T	–	13 (4.21%)	536 (21.41%)	2.20 × 10^− 14^	7.04 × 10^− 13^	–	Unknown
Intron	rs731934	35831008	c. C3481 + 45 T	–	21 (6.80%)	425 (16.97%)	1.71 × 10^−14^	5.48 × 10^−13^	–	Unknown

Unknown = Have not seen the relevant report at present. Statistical analysis was done by χ^2^ test

*Cy* C-terminal cytoplasmic, *Ig* immunoglobulin motif

The mutation c.G3315A (p.S1105S) in the exon 26 was the most common variant, the A allele frequency was 84.47% (261/309) in patients and was much higher than the data in controls (84.47% vs. 17.01%, *P* = 7.02 × 10^− 13^). The minor allele frequencies of the other three synonymous mutations (c.C294T [p.I98I], 30.10% vs. 6.27%, *P* = 7.51 × 10^− 30^; c.C2223T [p.T741 T], 15.21% vs. 1.24%, *P* = 2.50 × 10^− 25^; c.C2289T [p.V763 V], 32.04% vs. 12.14%, *P* = 1.61 × 10^− 13^) were also much higher in FSGS patients than in controls. The common variant c.C294T (p.I98I) located in Ig-1 domain, c.C2223T (p.T741 T) and c.C2289T (p.V763 V) in Ig-7 domain.

Two mutations were found in intron, c.C3286 + 36 T and c. C3481 + 45 T. The minor allele frequency (MAF) of c.C3286 + 36 T (4.21% vs. 21.41%, *P* = 7.04 × 10^− 13^) and c. C3481 + 45 T (6.80% vs. 16.97%, *P* = 5.48 × 10^− 13^) in FSGS patients were much lower than in controls.

### Missense mutations and splicing mutations

Seventeen missense mutations and one splicing mutation were detected in sporadic FSGS patients. A novel mutation c.G2638 T (p.V880F; Bonferroni-corrected *P* > 0.05) in exon 19 at a site of conserved Ig-8 domain, was detected only in one FSGS patient. The splicing mutation 35830957 C > T was also a novel variant and found only in one patient. The detail information of these mutations was listed in Table 2.

**Table 2:** The missense and splicing mutations detected in FSGS patients and compared with controls from 1000 Genomes Project

Exon	dbSNP144	Position	Transcript consequence	Protein consequence	Patients (*n* = 309)	Controls (*n* = 2504)	*P* value	Bonferroni-corrected *P* value	Nephrin domain	Initial phenotype
Missense										
1	rs191850409	35851824	c.C14T	p.T5M	3 (0.97%)	3 (0.12%)	0.020	> 0.05	Sp	Unknown
2	rs116617171	35851666	c.C65T	p.A22V	3 (0.97%)	9 (0.36%)	0.14	> 0.05	Ig 1	CNS [42], SRNS [43]
3	rs3814995	35851310	c.G349A	p.E117K	256 (82.85%)	418 (16.69%)	2.19 × 10^−14^	7.01 × 10^−13^	Ig 1	CNS [42], MCNS [41] SRNS/FSGS [43]
6	rs201822740	35849646	c.C616A	p.P206T	1 (0.32%)	1 (0.04%)	0.21	> 0.05	Ig 2	Unknown
7	rs201234008	35849314	c.C762A	p.H254Q	1 (0.32%)	1 (0.04%)	0.21	> 0.05	Ig 3	Unknown
7	rs115308424	35849273	c.G803A	p.R268Q	5 (1.62%)	11 (0.44%)	0.024	> 0.05	Ig 3	Unknown
8	rs113825926	35849107	c.C881T	p.T294I	1 (0.32%)	8 (0.32%)	1.00	> 0.05	Ig 3	MCNS [41], SRNS [43]
10	rs33950747	35848345	c.G1223A	p.R408Q	1 (0.32%)	42 (1.68%)	0.081	> 0.05	Ig 4	CNS [9, 25, 42], MCNS [41], FSGS [44]
11	rs28939695	35848142	c.G1339A	p.E447K	14 (4.53%)	16 (0.64%)	7.93 × 10^−7^	2.54 × 10^−5^	Ig 5	MsPGN/ FSGS/ SRNS [18], CNS [42], CNF [45]
14	rs114615449	35845496	c.G1802C	p.G601A	8 (2.59%)	9 (0.36%)	1.94 × 10^−4^	6.19 × 10^−3^	Ig 6	SRNS [18]
17	rs777418609	35843557	c.A2249G	p.E750G	1 (0.32%)	0	0.11	> 0.05	Ig 7	Unknown
17	rs115976159	358434987	c.C2309T	p.P770L	1 (0.32%)	0	0.11	> 0.05	Ig 7	Unknown
18	rs114896482	35842487	c.C2398T	p.R800C	10 (3.24%)	4 (0.16%)	1.49 × 10^−7^	4.78 × 10^−6^	Ig 7	SRNS [18, 40], MCNS [41]
19	–	35842149	c.G2638 T	p.V880F	1 (0.32%)	0	0.11	> 0.05	Ig 8	Novel
21	rs114849139	35839554	c.G2869C	p.V957vL	3 (0.97%)	9 (0.36%)	0.14	> 0.05	Fn	CNS [19, 42, 46]
24	rs4806213	35831699	c.A3230G	p.N1077S	2 (0.65%)	228 (9.11%)	7.67 × 10^−10^	2.45 × 10^−8^	Tm	SRNS [18], CNS [25, 42], CNF [47–49], MCNS [41]
24	rs199646631	35831655	c.C3274T	p.R1092C	1 (0.32%)	1 (0.04%)	0.21	> 0.05	Tm	FSGS [18]
Splicing	–	35830957	C > T	–	1 (0.32%)	0	0.11	> 0.05	–	Unknown

Unknown = Have not seen the relevant report at present. Statistical analysis was done by χ2 test

*Tm* transmembrane domain, *Fn* fibronectin type III motif, *Ig* immunoglobulin motif, *Sp* signal peptide

The mutation c.G349A, causing a substitution of glutamic acid by lysine at a site of Ig-1 domain (p.E117K), was the most common variants. The MAF of c.G349A was much higher in FSGS patients than in controls (82.85% vs. 16.69%, *P* = 7.01 × 10^− 13^). The MAFs of the other three missense mutations, c.G1339A (p.E447K) (4.53% vs. 0.64%, *P* = 2.54 × 10^− 5^) in Ig-5 domain, c.G1802C (p.G601A) (2.59% vs. 0.36%, *P* = 6.19 × 10^− 3^) in Ig-6 domain, c.C2398T (p.R800C) (3.24% vs. 0.16%, *P* = 4.78 × 10^− 6^) in Ig-7 domain, were also much higher in FSGS patients than in controls. One mutation, c.A3230G (p.N1077S), located in the transmembrane domain of nephrin. The MAF of c.A3230G (0.65% *vs.* 9.11%, *P* = 2.45 × 10^− 8^) was lower in FSGS patients than in controls.

Ten of the seventeen missense mutations had been reported previously to be related to CNS, SRNS, MCNS, as well as FSGS. But five of them weren’t significantly different between FSGS patients and controls after Bonferroni correction (Table 2). The remaining five missense mutations were mentioned above in this study, which distributed differently between FSGS patients and controls.

### Pathogenic mutations predicted by software

We used SIFT, Polyphen and MutationTaster to predict the pathogenicity of the 17 missense mutations in our study. As shown in Table 3, only five mutations, c.C616A (p.P206T), c.G1802C (p.G601A), c.C2309T (p.P770L), c.G2869C (p.V957 L), and c.C3274T (p.R1092C), were predicted to be disease-causing variants by the three different software at the same time. Of the five mutations, only c.G1802C (p.G601A) distributed differently between FSGS patients and controls. Specifically, c.G1802C (p.G601A) and c.G2869C (p.V957 L) have been reported before, both of them were found in Chinese patients. The former study [18] contained the data for the Sanger sequencing of five different podocyte-expressed genes in 38 Chinese children. NPHS1 showed a higher proportion of mutations, and c.G1802C (p.G601A) was illustrated as “likely pathogenic” with the prediction score. The c.G2869C (p.V957 L) was reported in a Chinese CNS family [19], and three heterozygous mutations were identified in NPHS1 gene. This mutation was predicted as “disease-causing variant” in our study.

**Table 3:** The pathogenic mutations predicted by three software for the non-synonymous mutations

Dbsnp144	Position	Transcript Consequence	Protein Consequence	SIFT	Polyphen	MutationTaster
rs201822740	35849646	c.C616A	p.P206T	Damaging	Probably damaging	Disease causing
rs114615449	35845496	c.G1802C	p.G601A	Damaging	Probably damaging	Disease causing
rs115976159	358434987	c.C2309T	p.P770L	Damaging	Possibly damaging	Disease causing
rs114849139	35839554	c.G2869C	p.V957 L	Damaging	Probably damaging	Disease causing
rs199646631	35831655	c.C3274T	p.R1092C	Damaging	Possibly damaging	Disease causing

Three different software are used to predict the likelihood of causing disease: SIFT, Polyphen and MutationTaster

The predicted results of the remaining 12 missense mutations were shown in Table 4. The novel mutation c.G2638 T, causing a conservative amino acid substitution of valine by phenylalanine, seemed to be a protective mutation.

**Table 4:** Pathogenicity predicted by three software for the non-synonymous mutations

Dbsnp144	Position	Transcript Consequence	Protein Consequence	SIFT	Polyphen	MutationTaster
rs191850409	35851824	c.C14T	p.T5M	Tolerated	Benign	Polymorphism
rs116617171	35851666	c.C65T	p.A22V	Damaging	Benign	Polymorphism
rs3814995	35851310	c.G349A	p.E117K	Tolerated	Probably damaging	Polymorphism automatic
rs201234008	35849314	c.C762A	p.H254Q	Tolerated	Benign	Polymorphism
rs115308424	35849273	c.G803A	p.R268Q	Tolerated	Benign	Polymorphism
rs113825926	35849107	c.C881T	p.T294I	Tolerated	Benign	Polymorphism
rs33950747	35848345	c.G1223A	p.R408Q	Tolerated	Probably damaging	Disease causing
rs28939695	35848142	c.G1339A	p.E447K	Tolerated	Probably damaging	Disease causing automatic
rs777418609	35843557	c.A2249G	p.E750G	Damaging	Possibly damaging	Polymorphism
rs114896482	35842487	c.C2398T	p.R800C	Tolerated	Benign	Polymorphism
–	35842149	c.G2638 T	p.V880F	Tolerated	Benign	Polymorphism
rs4806213	35831699	c.A3230G	p.N1077S	Damaging	Benign	Polymorphism automatic

Three different software are used to predict the likelihood of causing disease: SIFT, Polyphen and MutationTaster

## Discussion

Although NPHS1 was first demonstrated to be the causal gene for congenital nephrotic syndrome of Finnish type [20], subsequent studies have confirmed that it was also a causative gene or susceptibility gene for a variety of kidney diseases, such as SRNS, FSGS, minimal change disease with nephrotic syndrome (MCNS), IgA nephropathy, et al. [9, 11, 12, 21–24]. Among them FSGS was one of the most common causes of SRNS and a common type of genetically related kidney disease [18, 24, 25]. Most studies about genetic mutations in FSGS were based mainly on familial FSGS patients and lots of FSGS-causing genes have been identified [1, 5]. Especially in individuals presenting with FSGS or nephrotic syndrome before or at the age of 18 years old, the most common genes in which a mutation was found continues to be limited to only a few genes, including NPHS1 and NPHS2 [2, 23, 26–29].

Nephrin was located only in glomerular podocytes, which participated in intercellular junctions of mature podocyte and formations of the slit diaphragms [30]. It was demonstrated by lowering nephrin expression in an inducible model of nephrin deletion that normal nephrin expression was necessary for podocyte intercellular junction in the glomerulus [31]. A low level of nephrin expression could result in progressive proteinuria with glomerular hypertrophy and FSGS of glomeruli [31, 32]. Nephrin-knockdown mice developed more podocyte apoptosis and depletion after doxorubicin challenge [32]. NPHS1 knockout mice subjected to podocyte injury failed to recover from foot process effacement as well as the persistence of proteinuria [31]. Ephrin-B1, a membrane-bound protein, bound to and interacted with nephrin by immunoprecipitation assay [33]. The phosphorylation of ephrin-B1 enhanced the phosphorylation of nephrin and promoted the phosphorylation of c-Jun N-terminal kinase (JNK), which was required for ephrin-B1-promoted cell motility [33]. The interaction of nephrin and ephrin-B1 maintains the structure and barrier function of the slit diaphragm [33]. It suggested that nephrin was required to maintain slit diaphragm integrity and slit diaphragm-mediated signaling, and it played an important role for the maintenance of podocyte function, production of proteinuria and pathogenesis of FSGS.

So far, more than 220 different mutations have been described affecting most exons in NPHS1 [11]. These mutations have been found in various kidney diseases, however, the relationship between NPHS1 mutation and FSGS was relatively less explored [34–37]. Santin, et al. found that the mutations of NPHS1 gene were detected in patients with CNF, congenital FSGS, childhood FSGS, as well as adulthood FSGS [37]. A previous study reported that no pathogenic NPHS1 mutations were found in 33 FSGS patients with SRNS [36], but the study only explored the previously reported pathogenic mutations in a small FSGS sample. Another study indicated that gene interactions between NPHS1 and TRPC6 variants have important implications on post-transplantation FSGS [35]. In this study, we focused on sporadic FSGS patients to analyze the NPHS1 mutations. We identified 32 mutations in NPHS1 in a relatively large cohort of FSGS patients in China, including two novel mutations.

Twelve synonymous mutations, 17 missense mutations, one splicing mutation and two intron mutations were found in our study. The MAFs of four synonymous mutations (c.C294T [p.I98I], c.C2223T [p.T741 T], c.C2289T [p.V763 V], c.G3315A [p.S1105S]) and four missense mutations (c.G349A [p.E117K], c.G1339A [p.E447K], c.G1802C [p.G601A], c.C2398T [p.R800C]) were much higher in FSGS patients than the data in controls. The MAFs of a missense mutation (c.A3230G [p.N1077S]) and two intron mutations (c.C3286 + 36 T and c. C3481 + 45 T) were lower in FSGS patients than the data in controls. Interestingly, of the four synonymous and five missense mutations mentioned above, eight other mutations except synonymous mutation c.C294T (p.I98I) have been reported to be associated with SRNS, MCNS or FSGS (Tables 1 and 2). Five missense mutations, c.C616A (p.P206T), c.G1802C (p.G601A), c.C2309T (p.P770L), c.G2869C (p.V957 L), and c.C3274T (p.R1092C), were predicted to be disease-causing variants by three different software.

As listed in Table 1, several synonymous mutations were previously reported to be associated with SRNS, MCNS, or FSGS. But the pathogenesis was still unknown. For the missense mutations, although five mutations in our study were reported previously, the exact pathogenesis of these mutations remained unclear, and no appropriate animal model of these mutations has been established for further study. Nephrin contained eight Ig-like domains, a fibronectin type III-like module (Fn), a transmembrane domain and an intracellular domain [13, 38]. A previous study revealed that most mutations of NPHS1 were observed in Ig-2, Ig-4, and Ig-7 domains [39]. In our study, the differentially distributed mutations in FSGS patients mainly located in Ig-1, Ig-6, Ig-7, and C-terminal cytoplasmic regions. It was also consistent with the concept that Ig-like part of the intracellular domain remained to be crucial for nephrin function [29]. All of the results would be helpful for future pathogenic mechanism research.

## Conclusions

It was demonstrated in our study that the mutations of NPHS1 gene are common in Chinese sporadic FSGS patients. A comprehensive evaluation of NPHS1 mutations would be helpful for the management of FSGS. The mutations of NPHS1 gene could play an important role in the pathogenesis of FSGS, and we still needed more researches to clarify the pathogenesis of these mutations.

## Data Availability

The datasets used and/or analyzed during the current study are available from the corresponding author on reasonable request.

## Abbreviations

ACT4: Actin 4
CNF: Congenital nephrotic syndrome of the Finnish type
CNS: Congenital nephrotic syndrome
CsA: Cyclosporin A
Fn III: Fibronectin type III motif
FSGS: Focal segmental glomerulosclerosis
Ig: Immunoglobulin motif
IGSR: International genome sample resource
INF2: Inverted formin 2
MAF: Minor allele frequency
MCNS: Minimal change nephrotic syndrome
NPHS1: Nephrin
NPHS2: Podocin
OMIM: Online mendelian inheritance in man
SD: Slit diaphragm
SNV: Single nucleotide variant
SRNS: Steroid-resistant nephrotic syndrome
TRPC6: Transient receptor potential cation channel, subfamily C, member 6
WT1: Wilms tumor 1
